# Deacclimation-Induced Changes of Photosynthetic Efficiency, Brassinosteroid Homeostasis and *BRI1* Expression in Winter Oilseed Rape (*Brassica napus* L.)—Relation to Frost Tolerance

**DOI:** 10.3390/ijms23095224

**Published:** 2022-05-07

**Authors:** Julia Stachurska, Magdalena Rys, Ewa Pociecha, Hazem M. Kalaji, Piotr Dąbrowski, Jana Oklestkova, Barbara Jurczyk, Anna Janeczko

**Affiliations:** 1 The Franciszek Górski Institute of Plant Physiology, Polish Academy of Sciences, Niezapominajek 21, 30-239 Kraków, Poland; j.stachurska@ifr-pan.edu.pl (J.S.); m.rys@ifr-pan.edu.pl (M.R.); 2 Department of Plant Physiology, Faculty of Agriculture and Economics, University of Agriculture in Kraków, Podłużna 3, 30-239 Kraków, Poland; rrchilmo@cyf-kr.edu.pl (E.P.); barbara.jurczyk@urk.edu.pl (B.J.); 3 Institute of Technology and Life Sciences—National Research Institute, Falenty, Al. Hrabska 3, 05-090 Raszyn, Poland; hazem@kalaji.pl; 4 Department of Plant Physiology, Institute of Biology, Warsaw University of Life Sciences—SGGW, 02-766 Warsaw, Poland; 5 Institute of Environmental Engineering, Warsaw University of Life Sciences—SGGW, 02-766 Warsaw, Poland; piotr_dabrowski@sggw.edu.pl; 6 Laboratory of Growth Regulators, Faculty of Science, Institute of Experimental Botany of the Czech Academy of Sciences, Palacký University, Šlechtitelu 27, CZ-78371 Olomouc, Czech Republic; jana.oklestkova@upol.cz

**Keywords:** brassinosteroids, brassinosteroid insensitive 1, dehardening, delayed chlorophyll fluorescence, frost tolerance, homocastasterone, photosystem I, photosystem II, prompt chlorophyll fluorescence, stress tolerance

## Abstract

The objective of this study was to answer the question of how the deacclimation process affects frost tolerance, photosynthetic efficiency, brassinosteroid (BR) homeostasis and *BRI1* expression of winter oilseed rape. A comparative study was conducted on cultivars with different agronomic and physiological traits. The deacclimation process can occur when there are periods of higher temperatures, particularly in the late autumn or winter. This interrupts the process of the acclimation (hardening) of winter crops to low temperatures, thus reducing their frost tolerance and becoming a serious problem for agriculture. The experimental model included plants that were non-acclimated, cold acclimated (at 4 °C) and deacclimated (at 16 °C/9 °C, one week). We found that deacclimation tolerance (maintaining a high frost tolerance despite warm deacclimating periods) was a cultivar-dependent trait. Some of the cultivars developed a high frost tolerance after cold acclimation and maintained it after deacclimation. However, there were also cultivars that had a high frost tolerance after cold acclimation but lost some of it after deacclimation (the cultivars that were more susceptible to deacclimation). Deacclimation reversed the changes in the photosystem efficiency that had been induced by cold acclimation, and therefore, measuring the different signals associated with photosynthetic efficiency (based on prompt and delayed chlorophyll fluorescence) of plants could be a sensitive tool for monitoring the deacclimation process (and possible changes in frost tolerance) in oilseed rape. Higher levels of BR were characteristic of the better frost-tolerant cultivars in both the cold-acclimated and deacclimated plants. The relative expression of the *BRI1* transcript (encoding the BR-receptor protein) was lower after cold acclimation and remained low in the more frost-tolerant cultivars after deacclimation. The role of brassinosteroids in oilseed rape acclimation/deacclimation is briefly discussed.

## 1. Introduction

Oilseed rape (*Brassica napus* ssp.* oleifera* L.) is a major crop and is an important source of vegetable oil for the food, chemical and fuel industries. There are winter and spring cultivars of oilseed rape that differ with the season of growth and in crop yield. The yield of the winter cultivars is higher, and in Poland, winter oilseed rape is cultivated more often. The winter growth of the plants carries the risk of frost injuries that may lead to severe economic losses. Ice forms outside or inside a plant’s cells, causes membrane damage and initiates frost injures. However, the winter species have developed mechanisms that enable them to survive temperatures below 0 °C. A few weeks of cold acclimation (cold hardening usually at +2–+5 °C) improves the ability of plants to survive winter frost [1]. Cold acclimation causes many biochemical and physiological changes in the lipid and protein components of the cell membranes, soluble sugar content, osmotic potential and many other changes [2]. Sugar management is a particularly important element in the hardening process. The leaves of plants that have been cultivated in the cold retain an overexpression of the enzymes that are involved in sugar production and a have higher level of activity of the Calvin cycle enzymes compared to the leaves that have been grown before cold acclimation [3]. Well-cold-hardened plants of oilseed rape can survive at temperatures as low as about −20 °C. However, if the period of cold acclimation is interrupted by episodes of higher temperatures (deacclimation), the frost tolerance of plants decreases [1,4]. In recent years, such warm periods during late autumn have occurred more often due to climate change. Generally, deacclimation can occur when the temperature is higher than 9 °C, and in some circumstances, there can be an impulse to resume growth and development [1]. The rate of deacclimation depends on the temperature, plant species and plant genotype [5]. Warm periods have a negative effect on frost tolerance when they occur in late autumn, in winter and sometimes in early spring. Spring frost events increase the risk of frost injuries to plants [6]. During spring, when the temperature rises and then suddenly falls below zero, deacclimated plants are threatened, among others, because of a decrease in the amount of soluble carbohydrates that are necessary to survive frost [7]. Deacclimation has been well studied in woody plants [8], grasses [9] and the model plant *Arabidopsis* [3,10]. Although the physiological and biochemical changes that occur during deacclimation in oilseed rape are less understood, deacclimation definitely reduces the freezing tolerance in winter rape cultivars [4,11] which can cause stem elongation or even the development of buds [11]. After deacclimation, many of the biochemical/physiological parameters reached values that were on the level observed in the non-acclimated control [4]. Deacclimation was accompanied, among others, by a decrease in soluble sugar content (and osmotic potential), a decrease in the accumulation of the aquaporin protein (cellular water channels), and a decrease in the anthocyanin level, while there was an increase in the chlorophyll content, which was also associated with an increase in the efficiency of the light reactions of photosynthesis.

Photosynthesis is generally widely understood to be a highly sensitive indicator that mirrors the interaction of plants with environmental stressors, including the stress that is associated with an exposure to low temperatures. For example, cold slows down the Calvin cycle enzymes in *Arabidopsis* [12]. The cold-acclimated seedlings of *Pinus concorta* L. had an inhibited photosynthesis that was accompanied by a partial loss of the PSII reaction centers, which was indicated by the decreased levels of the reaction center D1 protein and the loss of chlorophyll. Conversely, the cold-acclimated winter wheat maintained a high level of photosynthesis and a chlorophyll content at the same level [13]. The cold-acclimated winter wheat cultivars had a CO_2_ assimilation and O_2_ evolution that were similar to or greater than the non-acclimated plants [14], and this was associated with, among others, an increased capacity for PSI cyclic electron transport [15]. Similar to many other physiological processes, the process of photosynthesis functions under the influence of phytohormones. Extensive studies have already been devoted to explaining the role of brassinosteroids (plant steroid hormones; BR) in photosynthesis [16]. A brassinosteroid-insensitive 1 (*bri1*) *Arabidopsis* mutant showed downregulation of the genes connected to the regulation of photosynthesis and was also characterized by reduced growth, lower photosynthetic activity and a disrupted PSII assemblage [17]. Studies of BR mutants of *Arabidopsis* that were conducted by [18] showed that brassinosteroids control the thylakoid membrane architecture and PSII function. *Arabidopsis cyp51A2* mutants that were defective in an early stage of the sterol biosynthesis pathway element, sterol 14α-demethylation, are lethal, and their genes, which are connected to the photosynthesis processes such as with the Rubisco large subunit, chlorophyll *a/b* binding protein and photosystem components, are downregulated and have reduced chlorophyll content and photosynthetic activity [19]. Conversely, BR deficiency can also lead to an increased accumulation of chlorophyll and the photosynthetic proteins that change the leaf color from green to dark green [20,21,22]. As for the effect of brassinosteroids on photosynthesis in plants that have been exposed to temperature stress (particularly low temperature), the knowledge is much more limited (reviewed in [23]). For example, in *Secale cereale*, BR stimulate the photoprotective mechanisms during a prolonged exposure to cold via the temporary suppression of the quantum efficiency of PSII, which is a consequence of energy dissipation in the form of non-photochemical quenching [24]. The duration of cold acclimation (three or six weeks) has a slightly different and cultivar-dependent effect on the regulation of the photosynthetic activity that is induced by BR in *Secale cereale* [25]. The brassinosteroid signaling pathways in plants are still being discovered, but an important element is the brassinosteroid membrane receptor protein (BRI1—brassinosteroid-insensitive 1) [26]. *BRI1* encodes a putative leucine-rich repeat receptor kinase. Mutations in *BRI1* result, among others, in semi-dwarfness or dwarfness in plants of *Arabidopsis thaliana* L. or barley [27,28]. The timing of flowering is also delayed in the BR-insensitive *bri1* mutants of *Arabidopsis* [29].

One of the most recent articles, in the section of the issue “expert views”, [8] clearly states that deacclimation after cold acclimation is “a crucial, but widely neglected part of plant winter survival”. Considering that the mechanisms of deacclimation are relatively poorly explained, especially regarding crop plants such as oilseed rape (where this phenomenon can cause severe injuries and economic losses), the main goal of our work was to study the deacclimation-induced changes in photosynthetic efficiency (PSI and PSII), brassinosteroid homeostasis and *BRI1* expression in winter oilseed rape. These changes were discussed in relation to the deacclimation-induced loss of frost tolerance. Moreover, we attempted to answer the question of whether the fluorescence measurements, which describe PSI and PSII efficiency, enable the potential changes in frost tolerance that are caused by the deacclimation process to be non-invasively predicted, which is well described in winter cereals [1] and can also be useful from the practical point of view in oilseed rape. Deacclimated plants were compared to plants that were non-acclimated and cold-acclimated. Ten cultivars were used for chlorophyll *a* fluorescence measurements and in frost tests. Based on the frost tests, four cultivars were selected and further examined for their BR content and *BRI1* accumulation.

## 2. Results and Discussion

### 2.1. Frost Tolerance

**Figure 1:**
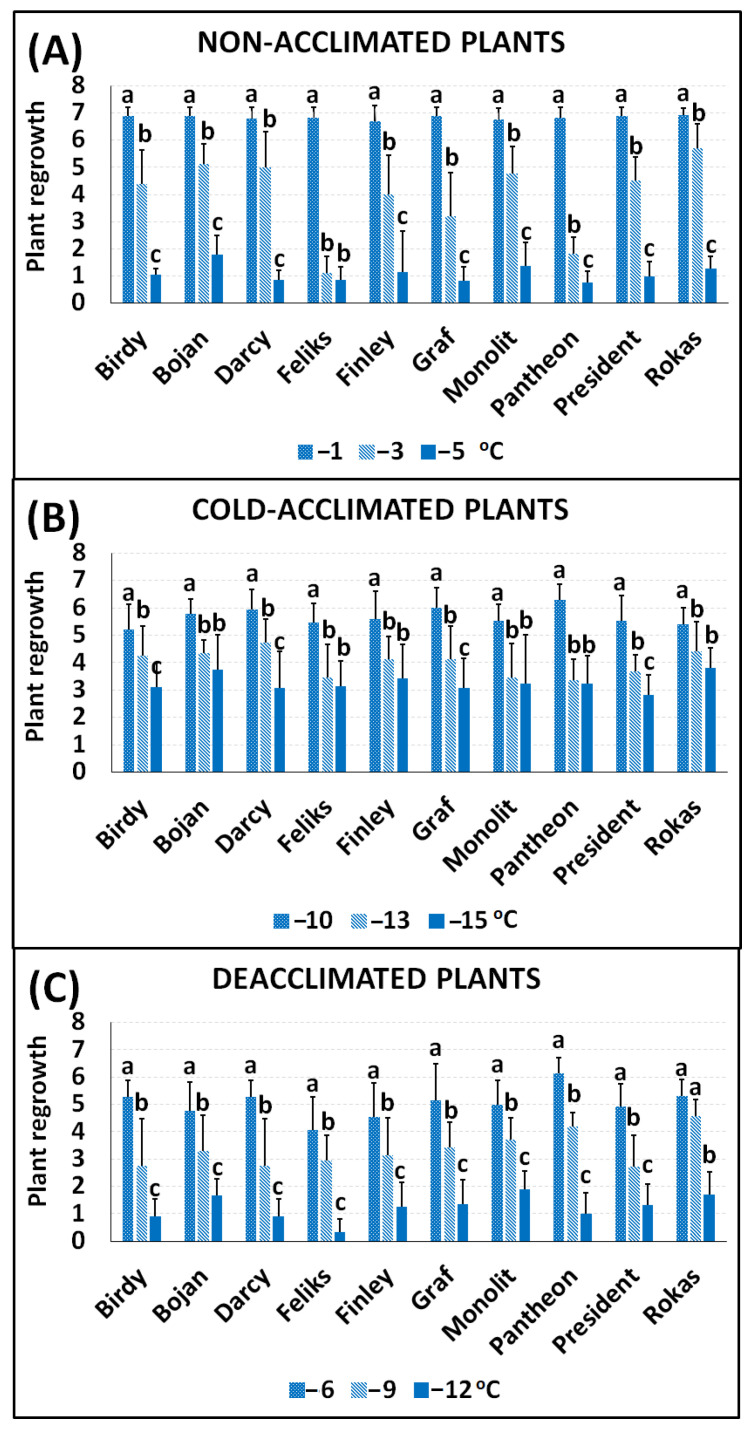
Frost tolerance of ten different cultivars of oilseed rape—Birdy, Bojan, Darcy, Feliks, Finley, Graf, Monolit, Pantheon, President and Rokas—that characterized the (**A**) non-acclimated plants, (**B**) cold-acclimated plants and (**C**) deacclimated plants. Frost tolerance based on the regrowth scale (0–7 points) after frost treatment (−1 to −16 °C); more detailed explanations of scale are in Section 3.3. Mean values ± SD that are marked with the same letters (separately for each cultivar) did not differ significantly at *p* < 0.05 according to Duncan’s test, *n* = 15.

**Figure 2:**
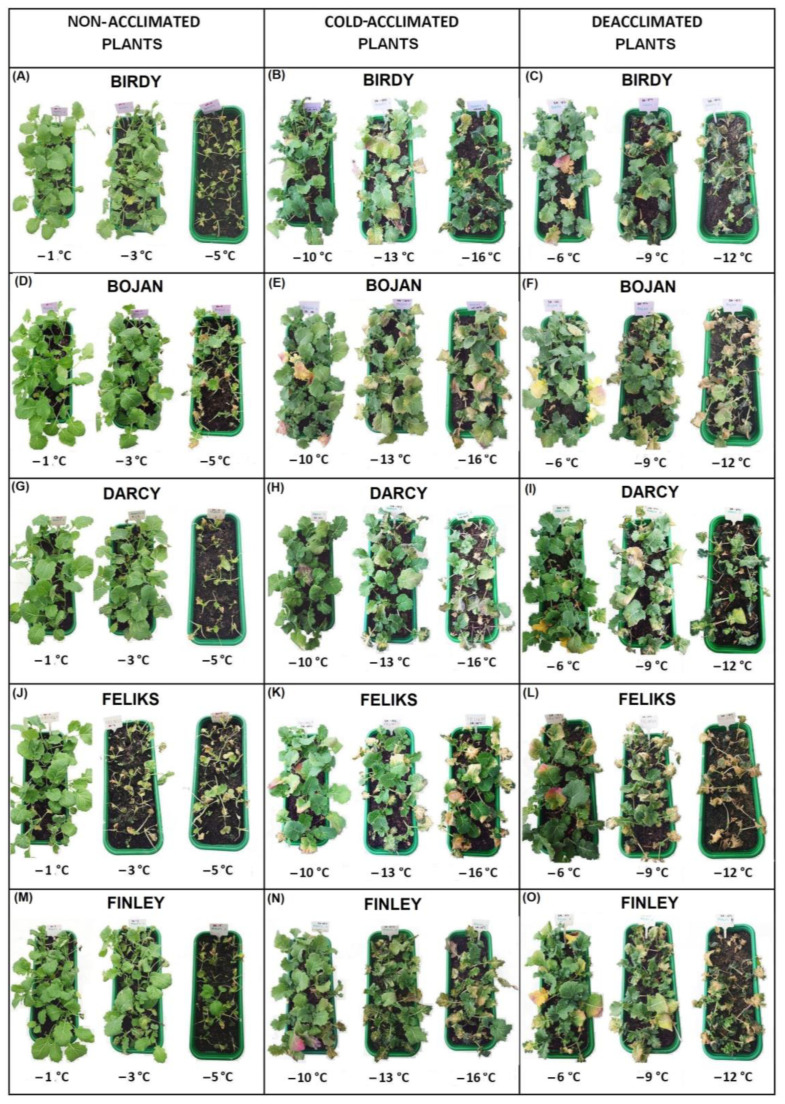
Plants of the non-acclimated, cold-acclimated and deacclimated oilseed rape (cultivars Birdy, Bojan, Darcy, Feliks, Finley) after exposure to frost. After frost treatment, the plants were left to regrow for two weeks at 12 °C. (**A**,**D**,**G**,**J**,**M**) non-acclimated plants; (**B**,**E**,**H**,**K**,**N**) cold-acclimated plants; (**C**,**F**,**I**,**L**,**O**) deacclimated plants.

**Figure 3:**
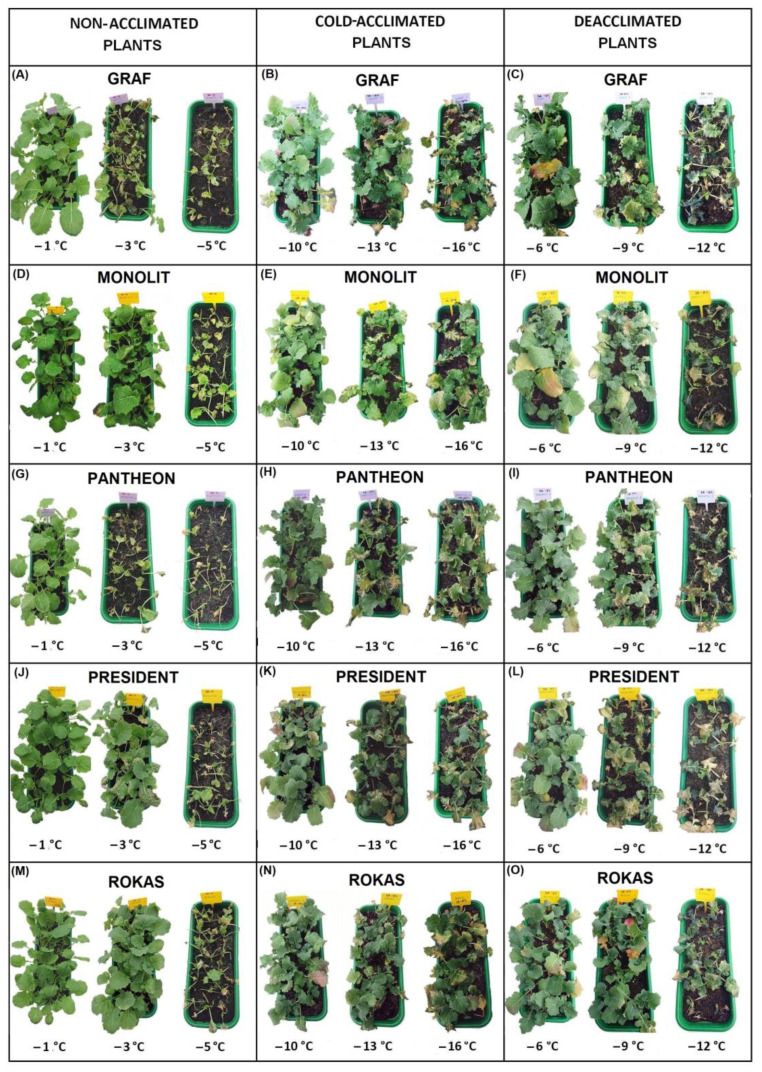
Plants of the non-acclimated, cold-acclimated and deacclimated oilseed rape (cultivars Graph, Monolith, Pantheon, President, Rokas) after exposure to frost. After frost treatment, the plants were left to regrow for two weeks at 12 °C. (**A**,**D**,**G**,**J**,**M**) non-acclimated plants; (**B**,**E**,**H**,**K**,**N**) cold-acclimated plants; (**C**,**F**,**I**,**L**,**O**) deacclimated plants.

The data about the frost tolerance of the plants were obtained by examining their regrowth—resumption of growth and the appearance of new leaves two weeks after freezing. A temperature of −1 °C did not seriously affect the non-acclimated (NA) plants, and all of the regrowth of the cultivars was on a level of between six and seven points (Figure 1A). The NA plants were barely able to survive a temperature of −5 °C, and they had the lowest frost tolerance at a level of below one point. Cold acclimation significantly improved the frost tolerance of the plants, which was expected. The cold-acclimated (CA) plants were able to survive temperatures of −13 and −14 °C, and regrowth was at a level of about three to four points (Figure 1B). The deacclimated plants (DA) were more sensitive to frost than the cold-acclimated plants (Figure 1C). Freezing the DA plants at a temperature of −12 °C enabled the regrowth at a level below two points in all of the cultivars. The frost tolerance of the deacclimated plants was, however, higher compared to the non-acclimated plants. A temperature of −5 °C caused regrowth in the non-acclimated plants at a level of about one point, while a temperature of −6 °C caused regrowth in the deacclimated plants at a level of about four to six points. The photographs of the oilseed rape plants that had been exposed to frost and then left to regrow (14 days at 12 °C) are presented in Figure 2 and Figure 3. Generally, our results are in agreement with the results that were obtained for oilseed rape by [4] and [11], where the authors found that deacclimation decreased the frost tolerance of oilseed rape. The current work, however, brings some new information due to the use of not one or two but ten cultivars with different traits. The estimated temperature that is required to reduce plant regrowth by 50% (RT50) was calculated for all of the cultivars, and this enabled the cultivars to be ranked (Table 1). As shown in Table 1, testing the ten different oilseed rape cultivars enabled us to observe that some oilseed rape cultivars with a high frost tolerance after cold acclimation such as Rokas also maintained a higher frost tolerance after deacclimation. The cultivar Feliks, which had a lower tolerance after cold acclimation, was characterized by low tolerance also after deacclimation. However, there were also cultivars that had a higher frost tolerance after cold acclimation but a decreased tolerance to frost after deacclimation (or opposite) as is clearly shown in Table 1. The higher basal frost tolerance (observed in the non-acclimated plants) did not result in a higher frost tolerance after deacclimation (see the cultivar President). Similarly, a low basal frost tolerance did not result in a low frost tolerance after deacclimation (see the cultivar Pantheon). Tolerance to deacclimation seems to be a cultivar-dependent trait. As tolerance to deacclimation, we generally understand that plants maintain a satisfactory level of frost tolerance (as much as possible similar to the level of frost tolerance acquired after cold acclimation) after warm periods that interrupt the process of cold hardening (acclimation) in autumn or after warm periods that appear in winter or even early spring.

Finally, based on the ranking (Table 1), four cultivars that differed in their frost tolerance were selected for a more detailed analysis of chlorophyll *a* fluorescence curves —Rokas, Feliks, Pantheon and President. The cultivar Rokas had a high frost tolerance of the NA, CA and DA plants. The cultivar Feliks had one of the lowest frost tolerances, especially in the non-acclimated and lowest tolerance in the deacclimated plants. The cultivar Pantheon had the lowest frost tolerance for NA plants, medium for CA plants, and high for DA plants. The plants of cultivar President had a relatively high frost tolerance if non-acclimated, a relatively low frost tolerance after cold acclimation and a low frost tolerance after deacclimation.

### 2.2. Prompt Chlorophyll a Fluorescence (PF)

**Figure 4:**
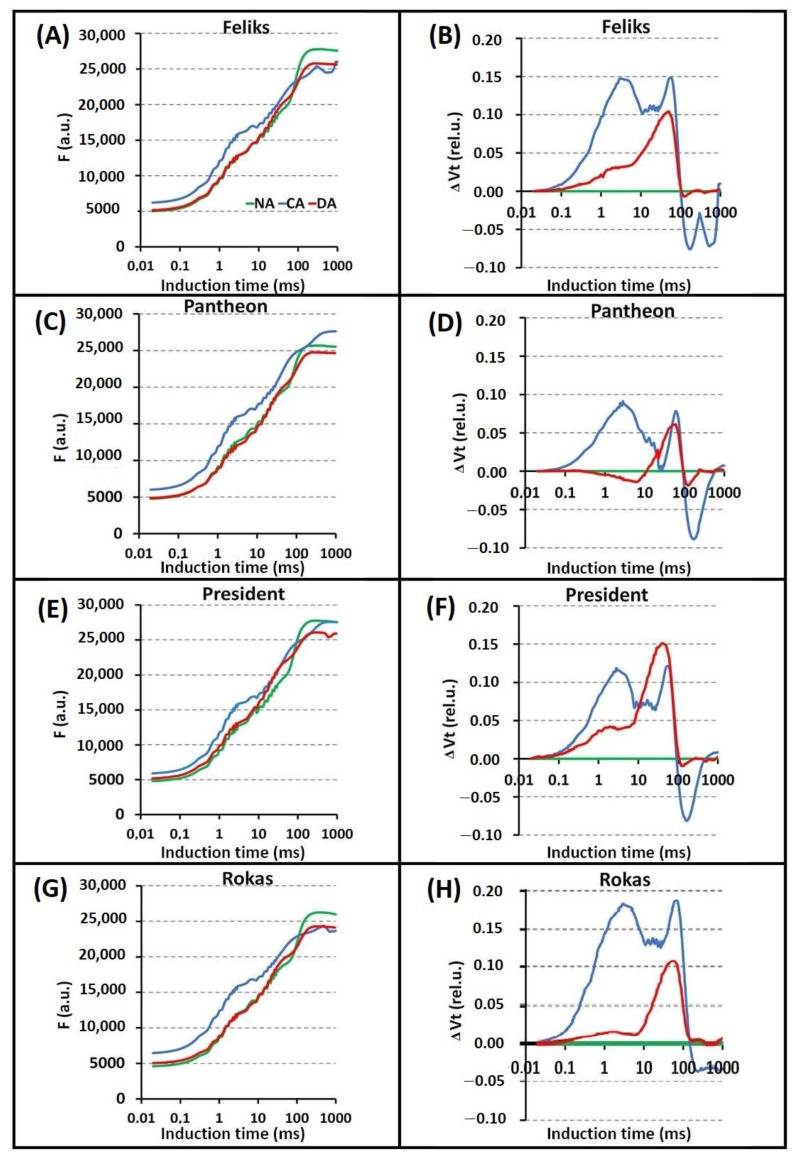
Induction curves of the chlorophyll *a* fluorescence (**F**) and the differential curves of _Δ_V_t_ (obtained by subtracting the control curve (non-acclimated plants—NA)) of oilseed rape. Plants that were not acclimated (NA), cold acclimated (CA) and deacclimated (DA). (**A**,**B**) cultivar Feliks; (**C**,**D**) cultivar Pantheon; (**E**,**F**) cultivar President; (**G**,**H**) cultivar Rokas. Statistical analysis of values of typical transient points for curves on figures (**A**,**C**,**E**,**G**) are given in Table S2.

**Figure 5:**
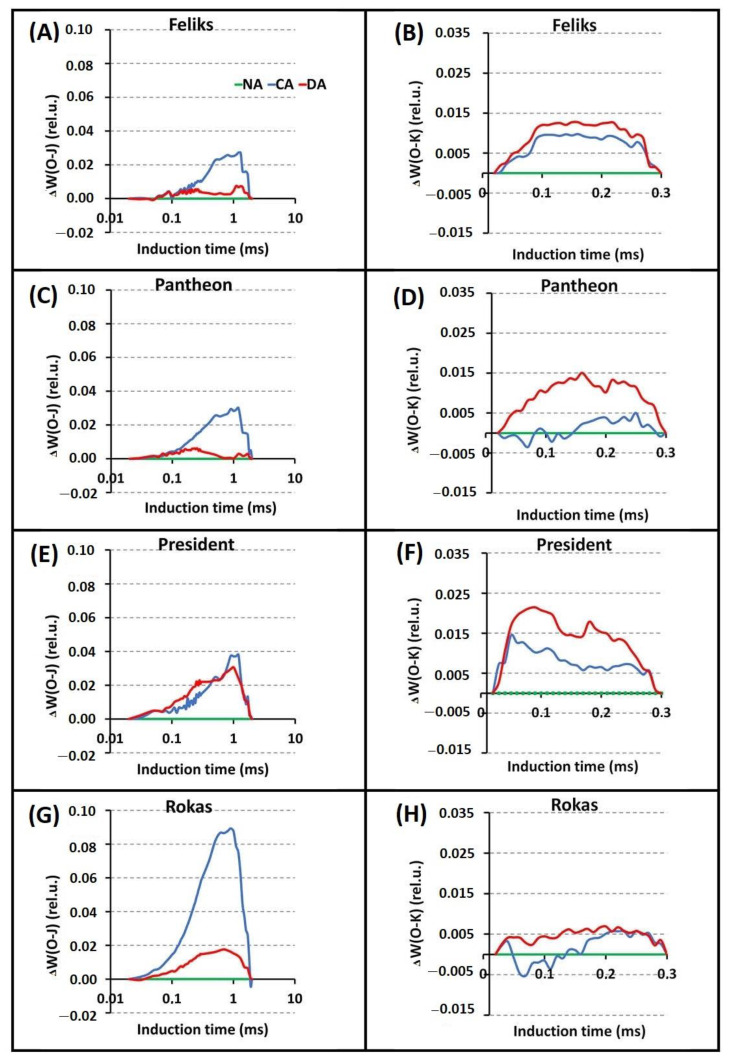
Differential curves of _Δ_W_(O-J)_ and _Δ_W_(O-K)_ (obtained by subtracting the control curve (non-acclimated—NA)) of oilseed rape. Plants not acclimated (NA), cold acclimated (CA) and deacclimated (DA). (**A**,**B**) cultivar Feliks; (**C**,**D**) cultivar Pantheon; (**E**,**F**) cultivar President; (**G**,**H**) cultivar Rokas.

**Figure 6:**
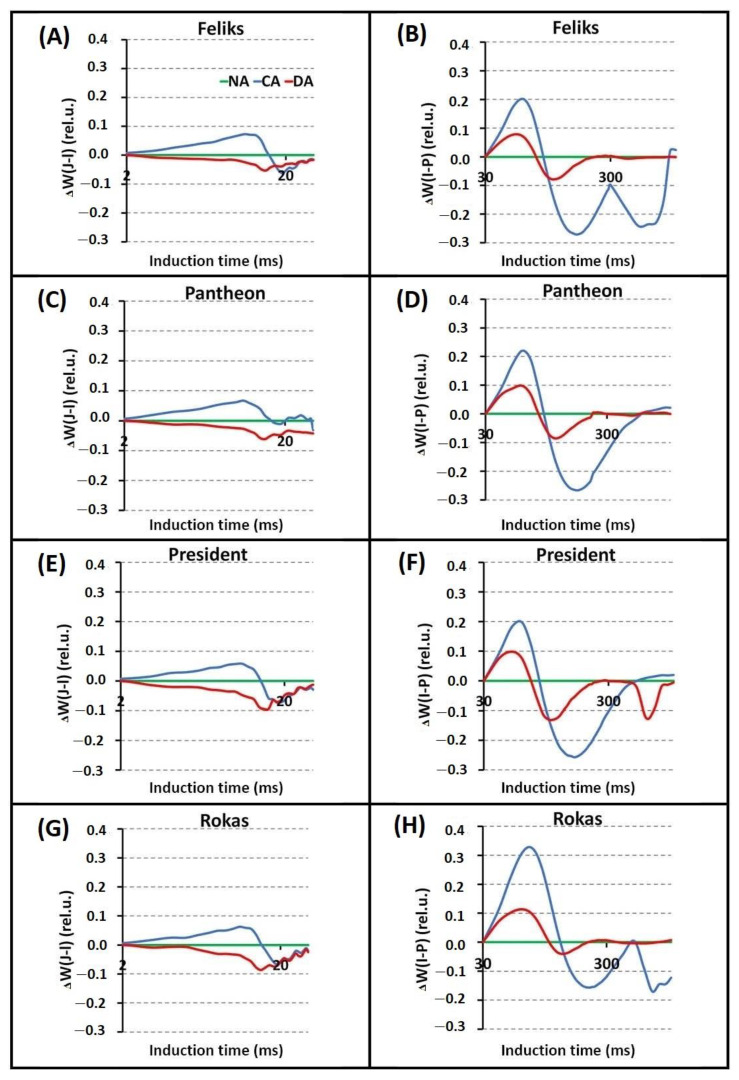
Differential curves of the _Δ_W_(J-I)_ and _Δ_W_(I-P)_ (obtained by subtracting the control curve (non-acclimated plants—NA)) of oilseed rape. Plants not acclimated (NA), cold acclimated (CA) and deacclimated (DA). (**A**,**B**) cultivar Feliks; (**C**,**D**) cultivar Pantheon; (**E**,**F**) cultivar President; (**G**,**H**) cultivar Rokas.

Based on the PF transients and their normalization, differences between the tested treatments (NA, CA, and DA) were significant in all cultivars (Figure 4, Tables S1 and S2). The cold-acclimation (CA) caused higher intensity of chlorophyll fluorescence than the NA and DA treatments, especially in the J–I phase. However, there were no significant differences between the cultivars. The differences between the cultivars were noticeable only after the normalization of the induction curves in the specific phases (O–J and O–K). The _Δ_W_(O–J)_ parameter revealed that the CA transient was most pronounced in the Rokas cultivar (Figure 5). The transient curves that were measured in DA plants had a lower intensity than those in the CA treatment. The _Δ_W_(O–K)_ parameter showed that the DA treatment had higher values compared to the CA treatment in all of the tested cultivars. During this phase, the Rokas cultivar showed the lowest intensity of chlorophyll fluorescence for both treatments. As can be seen in Figure 6, during the _Δ_W_(J–I)_ phase, the DA treatment caused a decrease in the intensity of chlorophyll fluorescence as well as in _Δ_W_(I-P)_, although there were no significant differences between the cultivars.

The increase in the _Δ_W_(O_-_K)_ might be connected with the inactivation of the oxygen-evolving complex (OEC) and/or the inhibition of the electron transport on the donor or acceptor side of photosystem II [30]. The _Δ_W_(O_-_K)_ also provides information about the grouping or connectivity—the relative position of the antenna complexes of the different RCs relative to each other [31]. A positive change in the course of the analyzed curve might indicate a greater distance between the PSII antennas and thus to less efficient energy exchange [32,33]. Conversely, the significant increase in the O–J step could be correlated with a reduction in the acceptor side of PSII [34], while _Δ_W_(I–P)_ provides information about the electron flow to the end electron acceptors of PSI [35].

**Figure 7:**
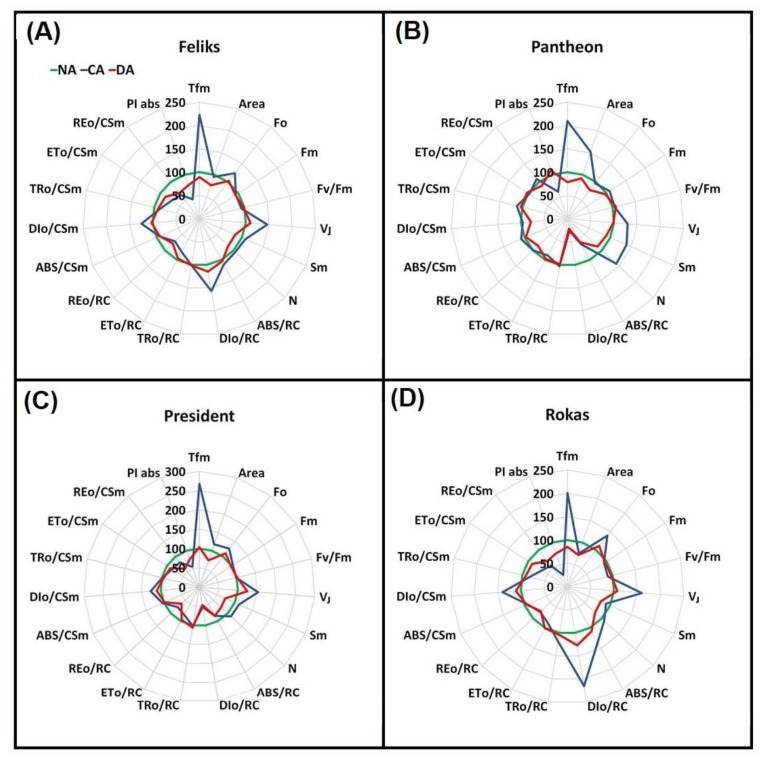
JIP—test parameters for the cold-acclimated (CA) and deacclimated (DA) plants of oilseed rape that were normalized to the values of the NA plants (non-acclimated control) as radar plots; NA is expressed as 100%. (**A**) cultivar Feliks; (**B**) cultivar Pantheon; (**C**) cultivar President; (**D**) cultivar Rokas. T_Fm_—time (in ms) to reach the maximal fluorescence intensity F_m_; Area—area above the curve; F_o_—minimal fluorescence, where all RC are open; F_m_—maximal fluorescence, where all RC are closed; F_v_/F_m_—maximum quantum yield of PSII photochemistry; V_J_—relative variable fluorescence at the J-step; Sm—normalized total area above the curve; N—amount of Q_A_ reduction from 0 to T_fm_; ABS—absorption flux (of antenna Chls); DI_o_—dissipated energy flux (at t = 0); TR_o_—trapping flux (leading to Q_A_ reduction); ET_o_—electron transport flux (further than Q_A_^−^); RE_o_—electron transport beyond PSI; RC—calculation in relation to reaction center; CSm—calculation in relation to excited cross section; PI_abs_—performance index (potential) for energy conservation from exciton to the reduction of intersystem electron acceptors. The description of fluorescence parameters is modified from [36] Detailed equations are also given in Table 2.

Looking at the selected values of the parameters of the JIP test, which are expressed as a radar plot (spider), it can also be concluded that there were differences between the three applied treatments (NA, CA and DA), and some differences were between the cultivars (Figure 7). Generally, deacclimation caused the changes that had been induced by cold acclimation to be reversed, which agrees with our earlier findings [4]. The pattern of the changes in the chlorophyll fluorescence parameters presented in Figure 7 (for four cultivars) shows that the response of the DA plants was more similar to the NA plants than to the CA plants. This phenomenon can also be well tracked based on the values of the F_v_/F_m_ and PI_abs_ and the parameters of the phenomenological fluxes and specific energy fluxes, which are presented in Table S1 for all ten tested cultivars. In all of the cultivars, most of these measured and/or calculated parameters (especially the F_v_/F_m_ and phenomenological fluxes), which are presented in Table S1, were similar in the NA and DA plants compared to the CA and DA plants.

Some cultivar-dependent changes were also observed. For example, the time it took to reach maximal fluorescence (T_fm_) significantly increased in the CA plants compared to the NA and DA plants (Figure 7). At the same time, the DI_o_/RC increased in the CA plants only in the Feliks and Rokas cultivars, while in the President and Pantheon cultivars, the values of these parameters were below the values that were observed for the non-acclimated control plants. The parameter PI_abs_ was usually lower in the CA and DA experimental variants and was similar in all four cultivars (the one exception was that the PI_abs_ parameter in cultivar Pantheon was similar to the one in the NA and DA plants).

The main visible effects of the acclimation/deacclimation procedures caused on the photosynthetic machinery were a decrease in the excitation energy migration toward the PSII reaction centers (an increase in the Fo in the CA compared to both the NA and DA plants), a slight fluorescence quenching in the CA and DA samples (decreased Fm values), a decrease in the electron transport rates on the PSII acceptor side and from the PQH_2_ pool to the PSI end acceptors, and deacclimation that partially restored the electron transport in the PSII acceptor side (the appearance of a J band).

**Figure 8:**
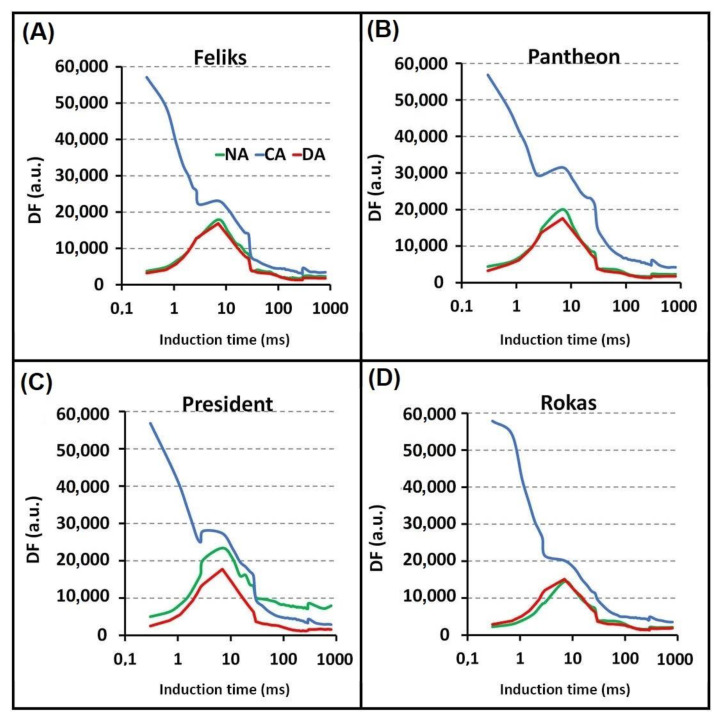
Delayed fluorescence induction curves for non-acclimated (NA), cold-acclimated (CA) and deacclimated (DA) oilseed rape. (**A**) cultivars Feliks; (**B**) cultivar Pantheon; (**C**) cultivar President; (**D**) cultivar Rokas. Statistical analysis of values of characteristic points of DF curves are given in Table S3.

The delayed chlorophyll fluorescence curves are presented in Figure 8. The beginning of the curves (before I_1_) in all of the tested cultivars that had been grown under the CA treatment had a high course. This might have been due to the cold-acclimation procedure, which induced structural changes in the photosynthetic machinery. Thus, it was reflected in the clear modifications of the DF induction curve.

Based on statistical analysis (Table S3), it can be stated that in all tested cultivars, the I_1_ point showed higher values under CA treatment as compared to NA. Simultaneously, DA treatment triggered the lowest value in all tested cultivars, except in Rokas. In Feliks and Rokas cultivars, the parameter I_1_-D_2_/D_2_ had lower values under CA treatment as compared to NA and DA treatments. The curves for the other cultivars had a similar trend in the I_1_, I_2_ and D_2_ points. Per the available literature, the changes in the I_1_ point might be related to two phenomena: (1) photochemical—accumulation of certain light-emitting states of the PSII reaction centers, and (2) non-photochemical—increase in the DF due to the electrical gradient formed by PSI when P700 is oxidized [37].

The I_2_ maximum (usually only a shoulder) is probably related to the prolonged reopening of PSII RCs by the electron transfer from the reduced Q_B_ to PQ before the full reduction of the PQ pool [38]. The changes in values of the D_2_ point correlate with the processes of reduction of the PQ pool, and the time when this point is reached can be an indicator of the reducing activity of the PSII complex [39].

The modifications of the first induction maximum reflect the formation and dissipation of the “light-emitting states” of PSII RC, i.e., the concentration of the precursors of the re-excited states of P680, which are the sources of the excitation energy for the DF emission. As a precursor to the micro-s DF, the PSII RC state P_680_^+^Q_A_^−^ has been proposed.

**Figure 9:**
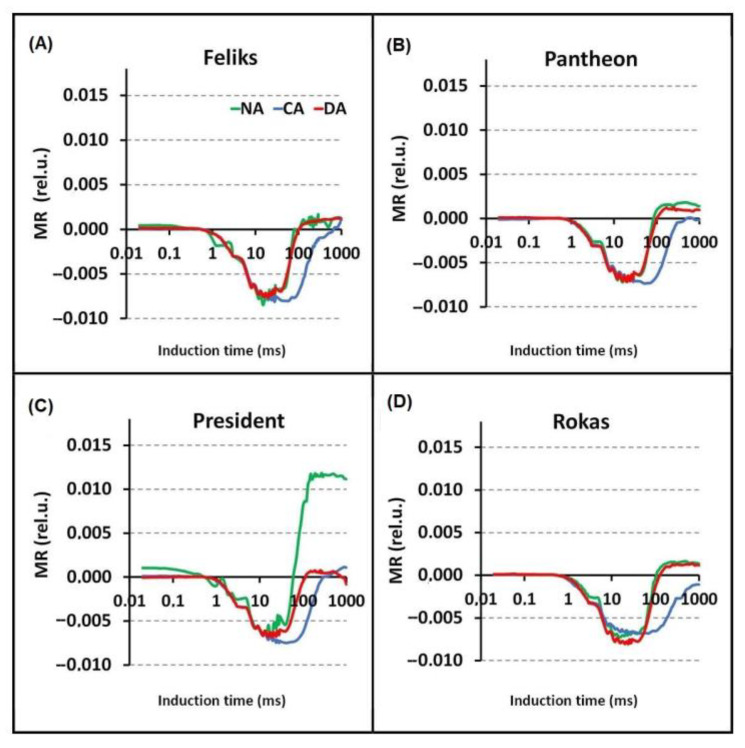
The kinetics of the modulated light reflection at 820 nm for the non-acclimated (NA), cold-acclimated (CA) and deacclimated oilseed rape. The data were normalized to the initial measured value of the signal. (**A**) cultivar Feliks; (**B**) cultivar Pantheon; (**C**) cultivar President; (**D**) cultivar Rokas. Statistical analysis of values of characteristic points of curves are given in Table S4.

The modulated infrared light reflection at the 820 nm (MR820) signals, which are primarily influenced by the rate of the electron flow from PSII to PSI, showed differences between the treatments and cultivars (in the CA) (Figure 9). The 820 nm reflection of the sample at the onset of the actinic illumination (MR_0_), the minimum (MR_min_) and the maximum (MR_max_) were analyzed (Table S4). There were significant changes between MR_0_ values, in particular treatments, and between cultivars. In all tested cultivars, the values of this parameter were lower under CA treatment as compared to NA. However, in Rokas, these changes were not significant. In addition, significant changes were noted in the MR_max_ parameter. In all tested cultivars, its values were lower under CA as compared to NA. The above-mentioned changes could have been the result of a limitation on the acceptor side of PSI [40,41]. Concluding remarks for all fluorescence measurements are given in Section 2.3.

### 2.3. Brassinosteroid Profile and the Transcript Level of BRI1

**Figure 10:**
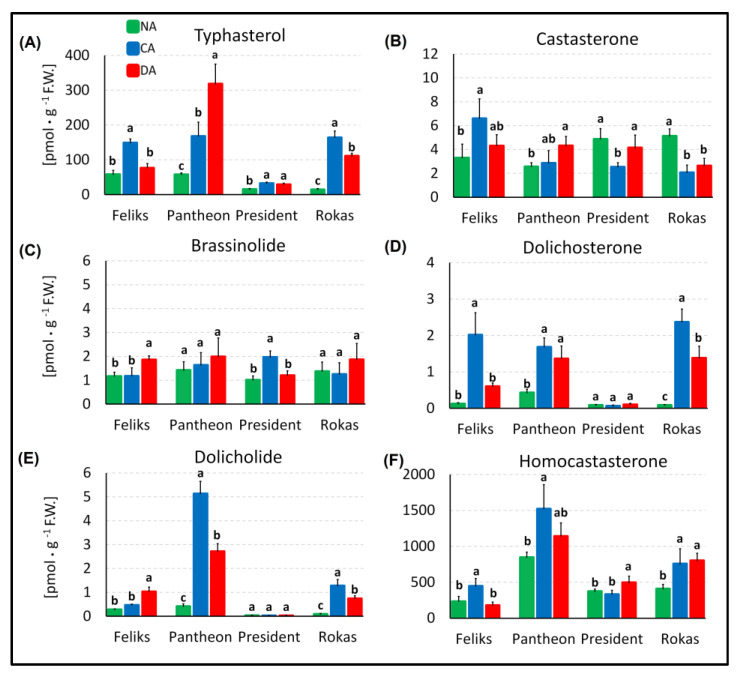
Content of brassinosteroids in the non-acclimated (NA), cold-acclimated (CA) and deacclimated (DA) oilseed rape (cultivars Feliks, Pantheon, President, Rokas). (**A**) Typhasterol; (**B**) castasterone; (**C**) brassinolide; (**D**) dolichosterone; (**E**) dolicholide; (**F**) homocastasterone (28-homocaststerone). Mean values ± SD that are marked with the same letters (separately for each cultivar) did not differ significantly at *p* < 0.05 according to Duncan’s test, *n* = 5.

The studies revealed the presence of typhasterol, castasterone, brassinolide, dolichosterone, dolicholide and homocastasterone (28-homocastasterone) in the leaves of oilseed rape (Figure 10). Homocastasterone and typhasterol were accumulated in the highest amounts. The other four BR were present in amounts that were even 1000-fold lower. Usually in oilseed rape, the content of brassinosteroids such as typhasterol, dolichosterone, dolicholide, and homocastasterone increases after cold acclimation, which agrees with previous results that had been obtained for winter wheat [42] and barley [21]. For these species was also reported an elevated level of BR after exposure to cold. Additionally, in winter wheat, there was correlation between the level of frost tolerance that was acquired after cold acclimation and brassinosteroid content. The more tolerant cultivars accumulated more BR (homocastasterone, teasterone) after cold acclimation. The dependency of the level of frost tolerance and BR content is generally observable in oilseed rape, although it is not as clear as in wheat. Rokas and Pantheon were more frost tolerant than Feliks or President after cold hardening (Table 1). After cold acclimation, the total BR content (sum of all measured BR), which was generally determined by the high content of homocastasterone and typhasterol (Figure 10A,F), was higher in Rokas and Pantheon (respectively, about 929 and 1703 pmol·g F.W.^−1^) and lower in Feliks and President (about 607 and 371 pmol·g F.W.^−1^, respectively). After deacclimation, Feliks and President definitely had the lowest frost tolerance (total BR content of about 265 to 531 pmol·g F.W.^−1^), while Rokas and Pantheon had the highest frost tolerance and about a 920 to 1471 pmol·g F.W.^−1^ content of total BR. It seems likely that a higher content of BR may be more desirable for better frost tolerance of oilseed rape in both the case of cold acclimated and, more importantly, in deacclimated plants. In this aspect, the findings of [43], who observed that the exogenous application of BR in *A. thaliana* (the same family as oilseed rape) resulted in a delay of generative development, are interesting. Since, as was mentioned in the Introduction, deacclimation often causes oilseed rape to develop too early, a naturally higher content of endogenous BR could be beneficial for preventing/limiting such phenomenon. This issue will need more detailed studies.

It is also worth mentioning a mechanism that was proposed by [44]. The authors described a model that had a BR concentration-dependent balance between growth and the stress response of plants. The model was focused on the interplay of BR, BRI1, ROS and ABA, where a higher concentration of BR was associated with a stress reaction, while a lower concentration was associated with a growth response. The role of brassinosteroids in the stress response in oilseed rape was also confirmed by [45]. The overexpression of the brassinosteroid biosynthetic gene *DWF4* increased stress tolerance (drought, high temperature) as well as resistance to some pathogens. Thus, our studies, which suggest that a higher BR content seems to be preferable for better frost tolerance, both after cold acclimation and deacclimation, are somewhat in agreement with these findings.

**Figure 11:**
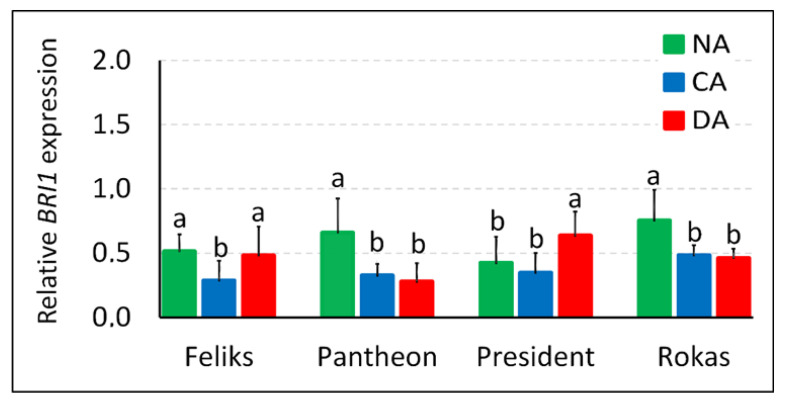
Relative transcript level of *BRI1* (brassinosteroid-insensitive 1, encoding the brassinosteroid membrane receptor) in the non-acclimated (NA), cold-acclimated (CA) and deacclimated (DA) oilseed rape (cultivars Feliks, Pantheon, President, Rokas). The transcript level was calculated relative to actin (endogenous reference gene). Mean values ± SD that are marked with the same letters (separately for each cultivar) did not differ significantly at *p* < 0.05 according to Duncan’s test, *n* = 3.

In the experiment, the transcript level of *BRI1* (an encoding receptor protein for brassinosteroids) was also studied. Cold hardening unambiguously lowered the accumulation of transcript *BRI1* in three of four of the tested cultivars (Figure 11). In one cultivar (President), the same tendency was noted, although it was statistically insignificant. After deacclimation, the accumulation of this transcript increased once again in the Feliks and President plants (and these cultivars simultaneously became more susceptible to frost (Table 1)). In Rokas and Pantheon, the accumulation of *BRI1* remained at the same low level as after cold acclimation, and these cultivars also maintained a high frost tolerance after deacclimation. Earlier, [46] observed that the *bri1-9 Arabidopsis* mutant had a higher tolerance to cold than the wild type, while the *BRI1*-overexpressing transgenic plants were more sensitive to cold. The higher tolerance to cold in the *bri1* mutant was associated with the constitutive activation of the stress-inducible genes. Conversely, according to [47], the *BRI1*-overexpressing plants of *Arabidopsis* had a higher frost tolerance after cold acclimation (compared to the wild type), while the *bri1-301* mutant was more susceptible to frost than the wild type. In our studies on oilseed rape, a higher level of the *BRI1* transcript was accompanied by a lower tolerance to low temperature. The results are then closer to the findings of [46], where the *BRI1*-overexpressing transgenic plants were more sensitive to low temperature. The amount of the transcript decreased after cold acclimation (together with an increase in frost tolerance), and simultaneously, the deacclimation-sensitive cultivars had increases in the transcript accumulation after a period of higher temperatures. We think that to obtain a more complete picture, the data about the accumulation of the BRI1 protein would be required in all of these studies.

Finally, a few comments should be made to the relationship between brassinosteroids and photosynthesis. The regulation of the photosynthesis process by BR is complex, and sometimes stimulatory or inhibitory activity of BR can be, which is dependent on the plant growth conditions or other factors [20,21,22,48]. In light of our results, it is interesting that the *bri1* mutant of *Arabidopsis* had a downregulation of the genes associated with the regulation of photosynthesis and that it was also characterized by reduced growth, lower photosynthetic activity and a disrupted PSII assemblage [17]. As was mentioned earlier, this *bri1* mutant also had a better tolerance to low temperature than the wild-type plants [46]. Consequently, in our oilseed rape, a lower accumulation of *BRI1* transcript was accompanied by a lower PSII efficiency during cold acclimation (compare Figure 11 and Table S1). The phenomenon was, however, not so clear after deacclimation. Despite the low level of *BRI1* in Rokas, for example, the PSII efficiency in this cultivar was higher compared to the efficiency observed after cold acclimation. Generally, the dependency of the brassinosteroid level, *BRI1* transcript (and protein level) and photosynthetic efficiency in cold-acclimated and particularly deacclimated plants would be an interesting matter for further, more in-depth studies.

To summarize and conclude, frost tolerance of oilseed rape after deacclimation was lower than frost tolerance after cold acclimation. Regarding tolerance to deacclimation, we generally understand that plants maintain a satisfactory level of frost tolerance (as much as possible similar to the level of frost tolerance acquired after cold acclimation) after warm periods that interrupt the process of cold hardening (acclimation) in autumn or after warm periods that appear in winter or even early spring. Tolerance to deacclimation seems to be a cultivar-dependent trait. As presented in the rankings (Table 1), some of the cultivars that had acquired a high frost tolerance after cold acclimation also maintained a high frost tolerance after deacclimation (Rokas). However, there were also cultivars that had a high frost tolerance after cold acclimation but partially lost it after deacclimation (Bojan) and/or cultivars with a lower frost tolerance after cold acclimation (compared to the other cultivars) but that better handled the deacclimation (Pantheon). Cold acclimation resulted in a particular pattern of changes in fluorescence; delayed fluorescence and deacclimation largely reversed those changes. The measurements of the various signals that are associated with photosynthetic efficiency (based on the prompt and delayed chlorophyll fluorescence signals) of plants can be a tool for monitoring the process of deacclimation (and potential changes in frost tolerance) in oilseed rape. However, we have to remember that although the fluorescence parameters of deacclimated plants resembled the control (not acclimated plants), they still had some remaining level of frost tolerance (higher than characteristic for not acclimated plants). This is probably because not all metabolic changes induced by cold acclimation (and responsible for frost tolerance after cold acclimation) were fully reversed during the seven days of deacclimation. Regardless, using drones, unmanned aerial vehicles (UAV) or satellites to monitor the changes in the fluorescence of crop fields, such measurements may enable the moment when plants are deacclimated to be estimated in late autumn/winter/early spring and therefore to be more sensitive to sudden frost. It can even enable precautions, such as spraying plants with regulators to improve their frost tolerance, to be undertaken. A higher content of brassinosteroids was more characteristic for the better frost-tolerant cultivars of oilseed rape in both the case of the cold-acclimated and deacclimated plants. The relative expression of the *BRI1* transcript (an encoding protein of the BR receptor) was lower after cold acclimation and remained low after deacclimation in the cultivars that were more tolerant to frost after deacclimation.

## 3. Materials and Methods

### 3.1. Plant Material

The study was conducted on ten cultivars of oilseed rape (*Brassica napus* ssp. *oleifera* L.), which are available for cultivation in Poland—Birdy, Bojan, Darcy, Feliks, Finley, Graf, Monolit, Pantheon, President and Rokas. Among the selected cultivars, nine are winter forms and only Feliks is a spring cultivar, which served as a kind of reference point for the lowest frost tolerance. Moreover, Birdy, Bojan, Darcy, Feliks, Finley, Monolit and Rokas are population cultivars. Graf, Pantheon and President belong to the hybrid (F1) cultivars.

Five of the ten cultivars that were used are characterized in the COBORU database (Development of Polish Official Variety Testing) in terms of plant height, the percentage of dead plants after winter 2015/16 and 2017/18 and the general condition of the plants after winter (nine-point scale of winter survival).

According to COBORU, the height of the plants of each cultivar is as follows: Birdy 139 cm, Bojan 160 cm, Feliks 121 cm, Graf 140 cm, Monolit 138 cm and Rokas 131 cm. As for other cultivars, we obtained general information from the breeder (Saatbau Poland), that the Pantheon and President cultivars are characterized by a tall plant, while the Darcy and Finley cultivars are semi-dwarf.

As was mentioned above, for some of the cultivars, data about winter survival are also available in the COBORU database. The percentage of dead plants after the winter of 2015/16 was 59% for Birdy, 41% for Graf, 21% for Monolit and 33% for Rokas. The percentage of dead plants after the winter of 2017/18 was 26% for Birdy, 19% for Monolit and 8.4% for Rokas. The general condition of the plants after the winter (on a nine-point scale) was 7.2 for Birdy, 8.0 for Bojan, 6.4 for Graf, 7.2 for Monolit and 7.0 for Rokas.

The seeds of the Bojan, Monolit and Feliks cultivars were derived from The Plant Breeding and Acclimatization Institute (IHAR), the National Research Institute in Strzelce (Poland). The seeds of the Darcy, Finley, Pantheon and President cultivars were derived from Saatbau (Poland). The seeds of Rokas were derived by Syngenta (Poland). The seeds of Birdy were derived from KWS (Poland), and the seeds of Graf were derived from the Obrol company (Poland).

### 3.2. Experimental Model/Plant Growth Conditions

Similar to the details that were described in an earlier work [4], the seeds were sown in Petri dishes in the dark at 24 °C (two days) for germination. The seeds were then transferred into 90 pots (40 × 15 × 15 cm; 18 plants/pot) with a soil mixture: the universal soil “Eco-Ziem Universal soil” (Eko-Ziem s.c., Jurków, Poland) pH = 5.5–7, the soil from the cultivation plots at the University of Agriculture (Kraków) and sand (2:1:1). The plants were cultured in a growth chamber in a controlled environment for three weeks (17 °C day/night (d/n); 12 h photoperiod). Three plants were removed from every pot to obtain a group of 15 plants of a uniform size. Next, for the pre-hardening, the plants were grown at 14 °C d/n (12 h photoperiod; two days); 12 °C d/n (8 h photoperiod; three days) and 10 °C d/n (8 h photoperiod, two days). Then, the temperature was lowered to 4 °C (8 h photoperiod, three weeks) for cold acclimation. Next, the plants were deacclimated at 16 °C/9 °C, d/n (8 h photoperiod, one week). The light intensity in the growth chamber was the same (at a canopy level of 300 µmol m^−1^s^−1^) during the entire experiment. Light source—LED lamps HORTI A (PERFAND LED, Trzebnica, Poland), which had been modified to emit light at a constant intensity and a blue light: red light spectrum (46%:54%). The experiment was conducted in the autumn/winter season (November/December 2020).

During the experiment, fluorescence measurements were taken three times: (1) after three weeks of growth at 17 °C (non-acclimated plants—control group), (2) after growth at 4 °C (cold-acclimated plants) and finally (3) in the deacclimated plants. The leaf samples for the analyses of the brassinosteroid content and transcript *BRI1* accumulation were collected at the same time points. After each time point, some of the pots with the plants were selected and placed in a freezing chamber to conduct the frost tests and to estimate the frost tolerance of the plants (for more details, see Section 3.3).

### 3.3. Testing of Freezing Tolerance of Cultivars

The test of the freezing tolerance was performed for each group of plants (treatments): non-acclimated, cold-acclimated and deacclimated plants (NA, CA and DA plants). The temperatures of freezing were selected based on the experience of the authors and were matched to the predicted level of frost tolerance of a specific group of plants. The non-acclimated plants were tested at −1, −3 and −5 °C; the cold-acclimated plants were tested at −10, −13 and −16 °C and the deacclimated plants were tested at −6, −9 and −12 °C. All of the pots were placed into the freezing chamber at 2 °C in darkness. Next, the temperature was decreased by 3 °C per hour until the required temperature (frost) was reached. The plants were kept at this temperature for 6 h. Afterward, the temperature was increased by 3 °C per hour to reach 2 °C, and the plants were transferred into the growth chamber at 12 °C (8 h photoperiod, 150 µmol m^−1^ s^−1^ light intensity). After two weeks of growth at 12 °C, the plant survival rate was evaluated using a visual score of:

0—a completely dead plant with no signs of leaf growth;

1—a dead plant without leaves (dead leaves had dropped). There was some small elongation of the leaves that were growing from the apical bud before it had died;

2—a plant that might not survive; there were leaves from the apical bud but they were small, discolored, or deformed;

3—a plant that has a chance of surviving but is badly injured (75% of the leaves are dead); leaf elongation occurred, new leaves are green but are thin and small;

4—a plant that has survived but has severe injuries; about 50% of its leaves are dead (brown and shriveled) or with necrosis spots; leaf elongation occurred, new leaves are green and healthy;

5—a plant that is alive, but some symptoms of freezing injuries are visible; about 25% of the leaves have visible damage such as drying around their edges, yellowing or with necrosis spots;

6—a plant where only 5% to 10% of the leaves show minor symptoms of freezing injuries such as small necrosis spots or dried leaves edges;

7—a plant with no symptoms of injury.

**Figure 12:**
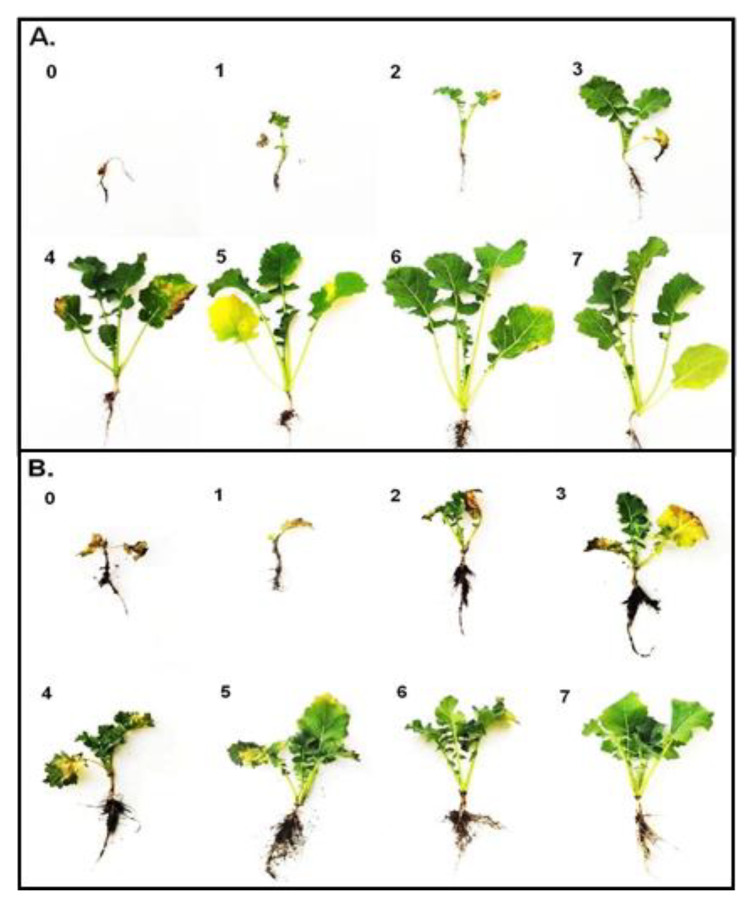
Scale showing the plant injuries after 14 days of their regrowth from the moment of frost exposure; (**A**) non-acclimated plants (**B**) cold-acclimated and deacclimated plants. See more details including an explanation of points 0–7 in Section 3.3.

Because there are some morphological/architectural differences between younger (non-acclimated) and older (cold-acclimated and deacclimated) plants that are more compact and shorter; photographs of exemplary plants at scale are presented separately for the non-acclimated plants (Figure 12A) and cold-acclimated and deacclimated plants (Figure 12B).

Finally, based on data of the frost tolerance for all three groups of plants (treatments NA, CA, DA), the estimated temperature that was required to reduce plant regrowth by 50% (RT50) was calculated as was described earlier in [48], and the cultivar ranking is presented in Table 1.

### 3.4. Chlorophyll a Fluorescence Measurements

Chlorophyll *a* fluorescence was measured using a MPEA+ Multi-Function Plant Efficiency Analyser (PEA, Hansatech Ltd., King’s Lynn, UK) for the analyses of photosystem I (PSI) and photosystem II (PSII). This apparatus measures prompt fluorescence, delayed fluorescence and modulated reflectance.

The leaves were adapted to dark for 30 min. using special clips. The measurements were taken in 15 replicates for each cultivar and treatment, using one leaf of an individual plant as one replicate. The measurements were always taken on the best-developed healthy leaves for a specific treatment (non-acclimated, cold-acclimated and deacclimated plants). Fluorescence measurements were taken for all ten cultivars, but more detailed analyses are presented in the article for four cultivars (Rokas, Feliks, Pantheon and President). Cultivars were selected based on calculated RT50 (Table 1).

The fluorescence signal was recorded with a maximum frequency of 105 points s^–1^ (each 10 ms) within 0–0.3 ms. In the next steps, the frequency of the recording decreased gradually, which resulted in the collection of 118 points within 1 s. The PF kinetics of the fluorescence were described by a fluorescence induction curve (fluorescence transient) in which its normalized analysis is known as the JIP-test [35,49]. The minimum curve is Fo, which is the initial fluorescence level measured at time 0.05 ms after actinic light, and maximum is Fm when the saturating light is applied to the leaf. There are also characteristic points between the minimum and maximum, which are labeled K (300 μs), J (2–3 ms), I (30 ms) and P (500–800 ms—1 s). The OJIP transients were induced by a short pulse (1 s) of saturating red light (650 nm and 3000 µmol s^−1^ m^−2^ of intensity) and were plotted on a logarithmic time scale.

In order to better visualize the effect of salt stress on the dynamics of the chlorophyll transients, the relative variable fluorescence was calculated. In the next stage, the differences in the relative variable fluorescence curves were calculated by subtracting the normalized fluorescence values (between the O and P steps) recorded in the control plants and plants under stress. The relative variable fluorescence intensity (V_t_) and the difference of the relative variable fluorescence intensity (ΔV_t_) were also calculated:

V_t_ = (F_t_ − F_o_)/(F_m_ − F_o_) (1)

ΔV_t, NA_ = V_t_,_CA or DA_ −V_t_, _control_ (2)

W_(O–J)_ = (F_t_ − F_o_)/(F_j_ − F_o_) (3)

W_(O–K)_ = (F_t_ − F_o_)/(F_k_ − F_o_) (4)

W_(J–I)_ = (F_t_ − F_j_)/(F_i_ − F_j_) (5)

W_(I–P)_ = (F_t_ − F_i_)/(F_p_ − F_i_) (6)

ΔW_t, NA_ = W_t_,_CA or DA_ − W_t_, _control_ (7)

The JIP-test was also used to recalculate the characteristic points of the photoinduced chlorophyll fluorescence transients to the specific parameters of the light phase of photosynthesis [49].

The DF and MR signals were recorded simultaneously with the PF. The characteristic points (I_1_, I_2_ and D_2_) of the DF curve were assessed according to [39]. The I_1_ point is the first maximum of a curve, the I_2_ point is the second maximum, and D_2_ is the second minimum of the curve. To better illustrate the changes of the DF induction curves, two ratios were calculated: (I_1_ − D_2_)/D_2_ and I_1_/I_2_.

The ratio MR_t_/MR_o_ was assessed from the MR signal. MR_t_ is the modulated 820 nm reflection intensity at time t, and MR_o_ is the value of the 820 nm reflection of a sample at the onset of the actinic illumination. MR_min_ is the minimum of the modulated 820 nm reflection intensity. MR_max_ is the maximum of the modulated 820 nm reflection intensity.

In order to better compare non-acclimated, cold-acclimated and deacclimated plants, the technical parameters were extracted from the curves of the fast fluorescence kinetic of chlorophyll *a.* The technical parameters and fluorescence parameters calculated based on them are presented on radar plots (Figure 7) for the four cultivars (Feliks, Pantheon, President and Rokas): T_fm_, Area, F_o_, F_m_, F_v_/F_m_, V_J_, Sm, N, ABS/RC, DI_o_/RC, TR_o_/RC, ET_o_/RC, RE_o_/RC, ABS/CSm, DIo/CSm, TR_o_/CSm, ET_o_/CSm, RE_o_/CSm and PI_abs_. T_fm_—time to reach maximal fluorescence (F_m_); Area—the area above the fluorescence induction curve from Fo to Fm; value is proportional to the size of the electron acceptors; F_o_—minimal fluorescence when all PSII reaction centers are open; Fm—maximal fluorescence when all PSII reaction centers are closed; F_v_/F_m_—maximum quantum yield of the photosystem II primary photochemistry; V_J—_relative variable fluorescence in step J (after 2 ms); Sm—normalized total area above the OJIP curve; N—turnover number (the number of Q_A_ reductions from time 0 to T_fm_); ABS—energy absorption by the antenna chlorophylls, TR_o_— trapping flux leading to Q_A_ reduction; ET_o_—electron transport flux (further than Q_A_^-^); DI_o_—dissipation of energy as heat; RE_o_—electron transport beyond PSI; PI_abs_—represents the general performance of PSII. Parameters (ABS, TR_o_, ET_o_, DI_o_) were expressed on the CS_m_ (the sample cross section) (phenomenological energy flues) and on the reaction center (RC) (specific energy fluxes). The detailed equations for particular parameters can be found in [36,50], and they are also presented in Table 2.

In Figure 7, the values for the non-acclimated plants are expressed as 100%, while the values given for the cold-acclimated and deacclimated plants are given as the percent changes compared to 100%. Original, average values of selected parameters (F_v_/F_m_, ABS/RC, TR_o_/RC, ET_o_/RC, DI_o_/RC, ABS/CSm, TR_o_/CSm, ET_o_/CSm, DI_o_/CSm, PI _abs_) for all ten cultivars are additionally presented in Table S1.

### 3.5. Analysis of the Brassinosteroid Content

BR analysis was performed as described in [51]. Plant leaves were powdered in liquid nitrogen, and then 60% acetonitrile was added. The samples were enriched by deuterium-labeled internal standards of brassinosteroids (25 pmol/sample, Olchemim s.r.o., Olomouc, Czech Republic). The samples were centrifuged, and the supernatant was passed through Discovery DPA-6S columns (Supelco, Bellefonte, PA, USA) and immunoaffinity (IA) columns (Laboratory of Growth Regulation, Olomouc, Czech Republic). The brassinosteroids were eluted from the IA columns using cold 100% methanol. The samples were dried and resuspended in 40 µL of methanol in order to measure the brassinosteroid content on a UHPLC using a tandem mass spectrometer (UHPLC-MS/MS) with an ACQUITY UPLC^®^ I-Class System (Waters, Milford, MA, USA) and a Xevo™ TQ-S MS triple quadrupole mass spectrometer (Waters MS Technologies, Manchester, UK). The detailed conditions of measurements are given in [51,52]. The analyses were performed in five repetitions, and each repetition included about 100 mg of fresh weight of the first and second leaf in the case of non-acclimated plants, the second and third leaf in the case of cold-acclimated plants and the fourth or fifth leaf of the deacclimated plants. This was because the period of growth of a culture was a total of about three months, and therefore, new leaves were developing systematically while the first leaves became senescent.

### 3.6. BRI1 Transcript Accumulation

The Quantitative Real Time PCR analysis for *BRI*1 expression was performed using QuantStudio 3 (ThermoFisher Scientific, Waltham, MA, USA). After collection, the leaf material was frozen in liquid nitrogen. RNeasy Plant Mini Kit (Qiagen, Hilden, Germany) was used for the RNA extraction and was enriched for mRNA according to the manufacturer’s protocol. Each RNA sample concentration and quality were determined spectrophotometrically (UV–Vis Spectrophotometer, Quawell, San Jose, CA, USA). Approximately 700 ng of RNA was subjected to a genomic DNA elimination, and immediately afterward, a reverse transcription reaction was performed (QuantiTect Reverse Transcription Kit, Qiagen, Hilden, Germany), according to the manufacturer’s protocol. The PCR primers and probes for *BRI*1 and actin *Brassica napus* genes (Table S5) were designed using Primer Express Software v.3.0.1 (Applied Biosystems by Life Technologies, Foster City, CA, USA). The PCR amplifications for *BRI*1 and *Actin* as the endogenous control genes were conducted in triplicate as was described by [53]. The PCR data were analyzed using QuantStudio Design and Analysis Software v.1.5.0. The relative standard curve method (Applied Biosystems) was used to calculate the relative gene expression. The *BRI*1 expression level was determined relative to the *actin*. The analyses were performed in three biological repetitions, and for each repetition, four technical repetitions were made. The leaf material (50 mg per sample) was collected as follows: fresh weight of first and second leaf in the case of the non-acclimated plants, the second and third leaf in the case of the cold-acclimated plants and the fourth or fifth leaf of the deacclimated plants. This was because the period of growth of a culture was a total of about three months, and therefore, new leaves were developing systematically while the first leaves became senescent.

## Figures and Tables

**Table 1:** Estimated temperature (°C) required to reduce plant regrowth by 50% (RT50). Ten cultivars of the non-acclimated, cold-acclimated and deacclimated oilseed rape were exposed to frost. The cultivars that were selected for a detailed analysis of the chlorophyll *a* fluorescence curves, analysis of brassinosteroid profile and *BRI1* expression are indicated with color.

Non-Acclimated Plants		Cold-Acclimated Plants		Deacclimated Plants	
Cultivar	RT50	Cultivar	RT50	Cultivar	RT50
Bojan	−3.68	Bojan	−13.54	Rokas	−8.91
Rokas	−3.34	Rokas	−13.50	Pantheon	−8.84
Monolit	−3.23	Darcy	−13.26	Graf	−8.41
President	−3.21	Graf	−13.24	Bojan	−8.40
Darcy	−3.12	Pantheon	−13.09	Monolit	−8.35
Birdy	−3.07	Monolit	−12.98	Finley	−8.31
Finley	−2.94	Feliks	−12.92	Darcy	−8.29
Graf	−2.69	President	−12.80	Birdy	−8.14
Feliks	−2.54	Finley	−12.76	President	−7.96
Pantheon	−2.35	Birdy	−12.44	Feliks	−7.63

**Table 2:** The description of fluorescence parameters, modified from [36].

T_Fm_	Time (in ms) to reach the maximal fluorescence intensity F_m_
Area	Area above the curve
F_o_	Minimal fluorescence, where all RC (reactive centers) are open
F_m_	Maximal fluorescence, where all RC are closed
F_v_/F_m_ = (F_m_ − F_o_)/F_m_	Maximum quantum yield of PSII photochemistry
V_J_ = (F_J_ − F_o_)/(F_m_ − F_m_)	Relative variable fluorescence at the J-step
Sm = Area/(F_m_ − F_o_)	Normalized total area above the curve
N = S_m_ M_o_ (1/V_J_) turn-over number Q_A_	Amount of Q_A_ reduction from 0 to T_fm_
ABS/RC = (1 − γ_RC_)/γ_RC_	Absorption flux (of antenna Chls) per RC
DI_o_/RC = (ABS/RC − TR_o_/RC)	Dissipated energy flux per RC (at t = 0)
TR_o_/RC = M_o_(1/V_J_)	Trapping flux (leading to Q_A_ reduction) per RC
ET_o_/RC = M_o_(1/V_J_)Ψ_o_	Electron transport flux (further than Q _A_^–^) per RC
RE_o_/RC = M_o_(1/V_J_)(1 − V_I_)	Electron transport beyond PSI
ABS/CS_m_ = F_m_	Absorption flux per excited cross section (CS_m_)
DIo/CS_m_ = (ABS/CS_m_) −(TRo/CS_m_)	Energy dissipation per CS_m_
TR_o_/CS_m_ = F_v_/F_m_ (ABS/CS_m_)	Energy flux for trapping per CS_m_
ET_o_/CS_m_ = (F_v_/F_m_)(1 − V_J_) F_m_	Energy flux for electron transport per CS_m_
RE_o_/CS_m_	Electron transport beyond PSI per CS_m_
PI_abs_ = γ_RC_/(1 − γ_RC_) × φ_Po_/(1 − φ_Po_) × Ψ_Eo_/(1 − Ψ_Eo_)	Performance index (potential) for energy conservation from exciton to the reduction of intersystem electron acceptors
I_1_ and I_2_	Maxima of DF induction curve
D_2_	Minimum of DF induction curve
MR_o_	Modulated 820 nm reflection intensity at time “0”
MR_min_	Minimum of modulated 820 nm reflection intensity
MR_max_	Maximum of modulated 820 nm reflection intensity
ΔMR_fast_	Fast phase (oxidation) of reflection intensity = MR_o_ − MR_min_
ΔMR_slow_	Slow phase (reduction) of reflection intensity = MR_max_ − MR_min_

## Data Availability

Not applicable.

## Supplementary Materials

The following supporting information can be downloaded at https://www.mdpi.com/article/10.3390/ijms23095224/s1.
