# Supplementary material for: Deacclimation-Induced Changes of Photosynthetic Efficiency, Brassinosteroid Homeostasis and BRI1 Expression in Winter Oilseed Rape (Brassica napus L.)—Relation to Frost Tolerance
Source: Int J Mol Sci. 2022 May 7;23(9):5224. doi: 10.3390/ijms23095224 (PMC9102500; doi:10.3390/ijms23095224)
Supplement: Supplementary file 1 [file ijms-23-05224-s001.zip › Table S1.pdf]

Table S1. Values of selected parameters of chlorophyll *a* fluorescence (Photosystem II efficiency) of non-acclimated (NA), cold-acclimated (CA) and deacclimated (DA) plants of oilseed rape (ten cultivars). *Average values ±SD marked with the same letters did not differ significantly at p≤0.05 according to Duncan's test, n=15.*

Treatment	Fv/Fm	ABS/RC	TRo/RC	ETo/RC	DIo/RC	ABS/CSm	TRo/CSm	ETo/CSm	DIo/CSm	P.I. ABS
Birdy NA	0.826±0.01 a	1.04±0.04 b	0.86±0.04 b	0.58±0.03 b	0.18±0.01 b	27783±793 a	22961±751 a	15676±848 a	4823±161 b	10.05±1.57 a
Birdy CA	0.711±0.06 b	1.44±0.34 a	1.01±0.19 a	0.51±0.03 c	0.43±0.17 b	23080±5330 b	16645±4954 c	8634±2923 c	6435±916 a	2.45±1.79 b
Birdy DA	0.806±0.01 c	1.09±0.12 b	0.88±0.08 b	0.62±0.04 a	0.21±0.04 a	24509±1509 b	19773±1424 b	13956±1335 b	4736±312 b	9.66±2.54 a
Bojan NA	0.822±0.01 a	1.01±0.07 b	0.83±0.05 b	0.59±0.02 a	0.18±0.02 b	27099±1553 a	22291±1388 a	15943±1183 a	4808±264 b	12.12±3.33 a
Bojan CA	0.726±0.11 b	1.61±1.07 a	1.06±0.33 a	0.50±0.08 b	0.54±0.79 a	25450±5352 ab	18914±5551 b	9822±3789 c	6536±993 a	3.20±2.64 c
Bojan DA	0.784±0.03 a	1.24±0.26 ab	0.97±0.16 a	0.62±0.04 a	0.27±0.09 ab	23803±1935 b	18647±1577 b	12138±1788 b	5156±815 b	6.67±3.46 b
Darcy NA	0.827±0.01 a	1.08±0.06 b	0.89±0.04 a	0.65±0.04 a	0.19±0.02 b	27700±1411 a	22914±1328 a	16735±953 a	4786±169 b	12.16±1.59 a
Darcy CA	0.746±0.08 b	1.33±0.47 a	0.96±0.22 a	0.53±0.05 b	0.37±0.27 a	25462±4296 b	19268±4906 b	11488±4200 c	6194±883 a	5.04±3.77 b
Darcy DA	0.807±0.03 a	1.15±0.23 ab	0.93±0.14 a	0.64±0.05 a	0.23±0.09 b	26274±1638 ab	21227±1758 a	14948±2221 b	5047±645 b	10.03±4.08 a
Feliks NA	0.824±0.02 a	1.11±0.10 a	0.92±0.07 a	0.65±0.04 a	0.20±0.04 b	27811±1605 a	22939±1661 a	16430±1870 a	4871±368 b	11.26±3.08 a
Feliks CA	0.763±0.05 b	1.23±0.37 a	0.92±0.20 a	0.52±0.06 b	0.31±0.18 a	26026±3502 b	19981±3711 b	11672±2873 c	6044±928 a	5.01±3.39 c
Feliks DA	0.805±0.02 a	1.15±0.15 a	0.92±0.10 a	0.63±0.04 a	0.23±0.05 b	25789±1244 b	20771±1159 b	14241±1278 b	5018±404 b	8.44±2.86 b
Finley NA	0.822±0.01 a	1.08±0.12 a	0.89±0.09 a	0.61±0.05 a	0.19±0.03 b	26049±1999 a	21418±1781 a	14712±1703 a	4631±401 b	10.20±3.31 a
Finley CA	0.775±0.03 b	1.11±0.25 a	0.86±0.17 a	0.48±0.03 c	0.26±0.09 a	24425±4102 ab	19049±3955 b	10963±3039 b	5376±318 a	4.88±2.36 b
Finley DA	0.810±0.01 a	1.02±0.07 a	0.83±0.05 a	0.58±0.02 b	0.19±0.02 b	23891±1744 b	19365±1485 b	13622±1376 a	4527±332 b	10.39±3.04 a
Graf NA	0.826±0.01 a	1.07±0.05 b	0.88±0.04 a	0.61±0.03 a	0.19±0.01 b	26979±1386 a	22291±1286 a	15400±1048 a	4688±196 b	10.08±1.57 a
Graf CA	0.753±0.05 c	1.20±0.20 a	0.90±0.09 a	0.52±0.04 b	0.31±0.12 a	25344±4126 ab	19266±4377 b	11406±3012 c	6078±509 a	4.37±2.22 b
Graf DA	0.801±0.02 b	1.15±0.25 ab	0.92±0.17 a	0.64±0.05 a	0.23±0.08 b	24749±1574 b	19828±1298 b	13950±1415 b	4921±632 b	9.67±3.50 a
Monolit NA	0.830±0.01 a	1.06±0.08 b	0.88±0.06 a	0.63±0.04 a	0.18±0.02 b	27204±1228 a	22567±1048 a	16178±1005 a	4637±276 b	12.21±3.50 a
Monolit CA	0.750±0.06 b	1.31±0.42 a	0.96±0.22 a	0.49±0.04 b	0.35±0.21 a	26217±3728 a	19852±4142 b	10735±3734 b	6365±924 a	3.78±2.59 b
Monolit DA	0.814±0.02 a	1.10±0.18 b	0.89±0.13 a	0.62±0.05 a	0.21±0.05 b	26485±1863 a	21576±1673 a	15082±1749 a	4909±449 b	10.46±4.23 a
Pantheon NA	0.754±0.21 a	1.89±2.69 a	0.88±0.07 a	0.62±0.05 a	1.01±2.64 a	25527±2180 b	19459±5838 a	13581±4173 a	6068±4533a	9.20±3.75 a
Pantheon CA	0.784±0.04 a	1.17±0.26 a	0.91±0.16 a	0.55±0.06 b	0.26±0.11 a	27648±4017 a	21799±4040 a	13302±2698 a	5850±545 a	5.69±2.74 b
Pantheon DA	0.808±0.02 a	1.08±0.20 a	0.87±0.13 a	0.60±0.04 a	0.21±0.07 a	24775±1634 b	20015±1493 a	14052±1553 a	4760±528 a	10.09±3.10 a
President NA	0.797±0.16 a	1.34±1.39 a	0.87±0.05 a	0.64±0.07 a	0.48±1.41 a	26608±5730 a	22058±5024 a	15879±3630 a	4550±728 c	11.61±3.02 a
President CA	0.788±0.04 c	1.13±0.27 a	0.88±0.16 a	0.52±0.04 b	0.25±0.11 b	27566±3035 a	21811±3253ab	13329±3186 b	5755±686 a	6.47±3.31 c
President DA	0.807±0.02 b	1.14±0.21 a	0.92±0.14 a	0.61±0.04 a	0.22±0.06 ab	26079±1255 b	21037±1099 b	14267±1564 b	5042±507 b	8.80±3.62 b
Rokas NA	0.829±0.01 a	1.01±0.03 b	0.84±0.03 b	0.60±0.03 a	0.17±0.01 b	26253±1201 a	21753±1077 a	15695±850 a	4500±158 c	12.62±1.99 a
Rokas CA	0.739±0.05 c	1.37±0.47 a	1.00±0.27 a	0.53±0.05 b	0.37±0.21 a	24597±4712 a	18328±4414 b	10128±2792 c	6268±887 a	3.40±1.97 c
Rokas DA	0.801±0.02 b	1.09±0.16 b	0.87±0.10 b	0.61±0.04 a	0.22±0.06 b	25097±2117 a	20121±1952ab	14134±1667 b	4976±448 b	9.50±3.19 b
