# Supplementary material for: Deacclimation-Induced Changes of Photosynthetic Efficiency, Brassinosteroid Homeostasis and BRI1 Expression in Winter Oilseed Rape (Brassica napus L.)—Relation to Frost Tolerance
Source: Int J Mol Sci. 2022 May 7;23(9):5224. doi: 10.3390/ijms23095224 (PMC9102500; doi:10.3390/ijms23095224)
Supplement: Supplementary file 1 [file ijms-23-05224-s001.zip › Table S2.pdf]

Table S2. Statistical analysis of values of typical transient points O, K, J, I and P of the chlorophyll *a* fluorescence induction curves presented on figure 3 A, C, E and G. Oilseed rape plants of cultivar Feliks, Pantheon, President and Rokas were not acclimated (NA), cold acclimated (CA) and deacclimated (DA). Average values ±SD marked with the same letters did not differ significantly at p≤0.05 according to Duncan's test.

Treatment	O	K	J	I	P
Feliks NA	4998±407 a	6018±525 a	11397±956 a	14044±1075 a	27848±1646 a
Feliks CA	6173±973 b	7486±1433 b	14227±2632 b	16692±2549 b	24589±6640 b
Feliks DA	5131±440 a	6160±665 a	11515±1083 a	14810±1351 a	25741±1283 a
Pantheon NA	4788±236 a	5729±374 a	11070±889 a	14060±1188 a	25569±2152 a
Pantheon CA	6020±598 b	7339±1010 b	14346±2174 b	17021±2432 b	27553±4097 a
Pantheon DA	4871±565 a	5776±892 a	10723±1468 a	14421±1809 a	24718±1646 a
President NA	4831±142 a	5751±176 a	11152±564 a	14073±789 a	27703±1196 a
President CA	5920±738 b	7197±1131 b	14237±2054 b	16716±2075 b	27564±3039 a
President DA	5181±552 a	6259±912 a	11812±1637 a	20720±1967 c	26070±1255 a
Rokas NA	4605±172 a	5461±259 a	10564±827 a	13556±1158 a	26217±1206 a
Rokas CA	6456±988 b	7895±1711 b	14468±3526 b	16831±3753 b	23544±6869 b
Rokas DA	5030±478 c	5899±695 a	10618±1205 a	14309±1445 a	24241±2116 a
