# Supplementary material for: Deacclimation-Induced Changes of Photosynthetic Efficiency, Brassinosteroid Homeostasis and BRI1 Expression in Winter Oilseed Rape (Brassica napus L.)—Relation to Frost Tolerance
Source: Int J Mol Sci. 2022 May 7;23(9):5224. doi: 10.3390/ijms23095224 (PMC9102500; doi:10.3390/ijms23095224)
Supplement: Supplementary file 1 [file ijms-23-05224-s001.zip › Table S3.pdf]

Table S3. Statistical analysis of values of characteristic points of DF curves measured for oilseed rape leaves and presented on figure 7. Oilseed rape plants of cultivar Feliks, Pantheon, President and Rokas were not acclimated (NA), cold acclimated (CA) and deacclimated (DA). Average values ±SD marked with the same letters did not differ significantly at $p \leq 0.05$ according to Duncan's test.

Treatment	I_1	I_2	D_2	I_1/I_2	$(I_1-D_2)/D_2$
Feliks NA	19100±882 a	4301±1892 a	1602±810 a	5.3±2.3 a	14.4±8.1 a
Feliks CA	25278±3945 b	5199±2702 a	3338±1491 b	6.2±3.1 a	8.2±4.6 b
Feliks DA	15968±2005 c	3452±2267 a	863±150 c	7.5±5.5 a	18.2±4.9 a
Pantheon NA	20114±602 a	3503±2321 a	1198± 169 a	8.1±4.1 a	16.3±2.3 a
Pantheon CA	32204±3972 b	10604±6322 b	2180±553 b	4.1±2.3 a	14.5±3.8 a
Pantheon DA	16502±2077 c	3443±2259 a	991±217 a	7.1±4.2 a	16.5±4.8 a
President NA	20758±1500 a	4371±2943 a	1369±376 a	7.2±4.6 a	15.3±4.6 a
President CA	28892±5533 b	8739±6833 b	1768±223 b	5.6±3.5 a	15.4±3.0 a
President DA	15472±2660 c	2278±1516 c	1144±425 a	10.1±6.2 a	14.7±7.6 a
Rokas NA	15096±961 a	3300±2219 a	956±247 a	6.9±4.6 a	15.5±3.3 a
Rokas CA	22298±3949 b	4388±1949 a	2594±431 b	5.7±1.9 a	7.9±2.4 b
Rokas DA	16644±886 a	2331±1494 a	910±202 a	10.1±5.7 a	17.9±3.3 a
