# Supplementary material for: Deacclimation-Induced Changes of Photosynthetic Efficiency, Brassinosteroid Homeostasis and BRI1 Expression in Winter Oilseed Rape (Brassica napus L.)—Relation to Frost Tolerance
Source: Int J Mol Sci. 2022 May 7;23(9):5224. doi: 10.3390/ijms23095224 (PMC9102500; doi:10.3390/ijms23095224)
Supplement: Supplementary file 1 [file ijms-23-05224-s001.zip › Table S4.pdf]

Table S4. Statistical analysis of the modulated infrared light reflection at the 820 nm (MR820) signals measured for oilseed rape leaves (curves presented on figure 8). Oilseed rape plants of cultivar Feliks, Pantheon, President and Rokas were not acclimated (NA), cold acclimated (CA) and deacclimated (DA). Average values ±SD marked with the same letters did not differ significantly at p≤0.05 according to Duncan's test.

Treatment	MR_0	MR_{min}	MR_{max}
Feliks NA	0.00044 a	-0.00874 a	0.00240 a
Feliks CA	0.00006 b	- 0.00813 a	0.00100 b
Feliks DA	0.00013 a	- 0.00764 a	0.00125 b
Pantheon NA	0.00002 a	-0.00720 a	0.00182 a
Pantheon CA	-0.00010 b	-0.00734 a	0.00009 b
Pantheon DA	0.00009 a	-0.00715 a	0.00725 c
President NA	0.00101 a	-0.00673 a	0.0128 a
President CA	0.00011 b	-0.00749 a	0.00114 a
President DA	-0.00001 c	-0.02026 b	0.00077 b
Rokas NA	0.00005 a	-0.00726 a	0.00163 a
Rokas CA	0.00006 a	-0.00683 a	0.00010 b
Rokas DA	0.00012 a	-0.00847 a	0.00136 c
