# Supplementary material for: Deacclimation-Induced Changes of Photosynthetic Efficiency, Brassinosteroid Homeostasis and BRI1 Expression in Winter Oilseed Rape (Brassica napus L.)—Relation to Frost Tolerance
Source: Int J Mol Sci. 2022 May 7;23(9):5224. doi: 10.3390/ijms23095224 (PMC9102500; doi:10.3390/ijms23095224)
Supplement: Supplementary file 1 [file ijms-23-05224-s001.zip › Table S5.pdf]

Table S5. Genes, sequence origins and the designed primers and probes used in the study.

Gene name	GenBank ID	Forward primer	Reverse primer	Probe
*BRI*1	JX871217.1	GATGTTCAAGCA ATCCGGGAAAA	TCTTTCTTCATCCCGTC GTTTTTTATGTA	FAM-TCGCTGTGAATTTCA-NFQ
Actin	AF111812.1	GCTATCCTCCGT CTCGATCTC	GTGGTGAACATGTACC CTCTCT	FAM-ACCTCACTGATTCCC-NFQ
